# Insights into Exosome in the Intervertebral Disc: Emerging Role for Disc Homeostasis and Normal Function

**DOI:** 10.7150/ijms.75285

**Published:** 2022-09-25

**Authors:** Xin Zhao, Benchi Xu, Wei Duan, Le Chang, Rui Tan, Zhen Sun, Zhengxu Ye

**Affiliations:** Department of Orthopedic, Xijing Hospital, Fourth Military Medical University. Western Changle Road, Xi'an, 710032, Shannxi Provence, P. R. China.

**Keywords:** exosome, intervertebral disc, low back pain, stem cells, intervertebral disc degeneration

## Abstract

Low back pain (LBP) is a chronic condition that causes great individual suffering and economic burden. The major contributor of LBP is intervertebral disc degeneration (IDD), which is caused by a spectrum of homeostasis alteration, including the apoptosis of nucleus pulposus (NP) and annulus fibrosus (AF) cells, degradation of extracellular matrix (ECM), calcification of cartilaginous endplates (CEP) and so on. Currently, the therapeutic strategy for IDD includes conservative and surgery treatment. Nevertheless, none of them could reverse the progressive destruction of the intervertebral disc. Hence, it is pivotal to pursue a new therapeutic approach. Exosomes, nano-sized substances with diameters of 30-150 nm, can be synthesized and secreted by various types of cells. They play an important role in intercellular communication. Increasing evidence implicates that exosomes could impact the intracellular transcription activities, thereby inhibiting or accelerating the proliferation and apoptosis of cells. Thus, it is a new therapeutic source for IDD. This review chiefly focuses on generalizing and clarifying the roles of exosomes in the onset and deterioration of IDD, and their therapeutic potential.

## Introduction

Low back pain (LBP) is a very common symptom which not only leads to individual suffering, but social and economic burden 1. The direct economic expenditure of LBP and health care is estimated to be around $87 billion in the US a year 2. In Korean, LBP is the second disease in socioeconomic burden and expenditure for management 3. The highest incidence of LBP happens to the people over 44 years old 2. With the average life expectancy longer than ever, it is urgent to discover the safest and most efficacious treatment for LBP.

The etiology of LBP is multifactorial, and the major contributor is considered to be intervertebral disc degeneration (IDD) 4-8. The risk factors of IDD mainly include age 9, genetic predisposition 10-12, mechanical damage 13, high body mass index, obesity 14 and so on 15. Current therapeutic strategies for IDD contain conservative and surgical options, but the outcomes are not invariable pleased 16. Besides, patients might not rehabilitate after surgery. Therefore, it is urgent to find a new therapeutic means which could reverse the degeneration of intervertebral disc (IVD) and free the patients from soreness.

Exosome was first visualized and named in the 1980s, which was considered to be the dumpster of cell and a way to dispose of unwanted molecules 17-20. However, recent studies gradually find that the function of exosome is far more than just delivering unwanted molecules 21, 22. It, as a tiny membrane vesicle which carries miRNA, mRNA and protein, is the mediator of intercellular communication and plays a pivotal role in the onset and deterioration of IDD 23, 24. This review tries to summarize the observations about various cells-derived exosome and their function in the outset and development of IDD. In addition, we also discussed the exosome-based biological therapy of IDD.

## Intervertebral Disc Degeneration

### Structure of IVD

IVD, derived as the tissue that bears load of spine, constitute one-third of the height of spine 25, 26. As the largest avascular structure of the body, IVD is composed of nucleus pulposus (NP), annulus fibrosus (AF) and cartilaginous endplates (CEP) 27. NP, a high-pressured and hydrophilic structure, is composed of water, cell and extracellular matrix (ECM). The ratio of water in NP is around 70-90%, which indicates that NP is the highest water content part of IVD 28; chondrocyte-like cells and notochordal cells are the main part of NP cells (NPc), which could secrete cartilage-like ECM components; ECM, made up of collagen, elastin, proteoglycans, and glycoproteins, participates in the metabolism and mechanical function of NP. AF, a ring-shaped disc of fibrosus connective tissue, is the fundamental load-bearing complex of IVD. It is composed of outer and inner annulus. The outer annulus principally comprises fibroblasts and the collagenated lamella; the inner annulus mainly consists of chondrocyte-like cells. CEP, a cartilaginous structure, forms the interface between adjacent vertebral segments 29, 30. Due to the avascular feature of IVD, the essential nutrients and oxygen all supplied via the concentration gradients exchange in CEP.

In IVD, the volume of cells is only 1%, with the average cells density is nearly 6000/mm^3^. NP cells density is 5-103/mm^3^, AF cells density is 9-103/mm^3^ 31, 32. The rare density of IVD cells signifies that when the IVD is impaired, the restoration rate is slow.

### Pathogenesis of IDD

A spectrum of pathogenic factors and intercellular effects participate in the IDD progression 33. For instance: superfluous mechanical stress; excessive oxidative stress; adverse inflammatory cytokines 34-40. Besides, diabesity and high body mass index which might change the micro-vasculature of CEP and adjacent vertebral body also influence the nutrient supply into IVD, thus accelerating the development of IDD 39-41.

The metabolic dysregulation of NPc leads to the suppression of synthesis of ECM; the lamellae in the AF become irregular; and the calcification of the tiny pores within CEP decreases the supply of nutrition and oxygen 42-45. All of which further aggravate the degeneration of IVD, unbalance the homeostasis of IVD, thus inducing the senescence and apoptosis of NPc and strengthening the permeation of inflammatory cytokines 46-50. Significantly, AF cells are sensitive to mechanical pressure. The alteration of ECM composition and senescence of NPc leading to exceeding load onto AF, thus inducing the apoptosis of AF cells and rupture of AF 51, 52. In addition, vascellums and nerves grow into IVD, causing pain 53-57. Taken together, these multifactorial changes eventually cause the collapse of IVD structure.

### Current Therapy of IDD

Currently, the therapeutic strategy for IDD mainly includes conservative and operative options 16. The conservative therapeutic strategy includes non-pharmacological and pharmacological therapy. The operative therapeutic strategy includes discectomy with/without fusion and total disc replacement (TDR) 58, 59. However, in conservative therapeutic strategy, the effect of non-pharmacological therapy is ambiguous; pharmacological therapy might cause drug addiction. In operative therapeutic strategy, complications after discectomy surgery might inevitable; the area of application of TDR is harsh 60.

The conservative and operative therapeutic strategies mainly aimed at relieving symptoms rather than reversing pathogenic progression of IDD. Therefore, it is essential to explore a novel therapeutic strategy which could delay and/or reverse the pathogenic process of IDD. Significantly, the stem cells and stem cell-derived exosome (SC-exo) based therapy is a newly developing non-invasive therapeutic approach. It has potent ability to suppress the IDD progression 61, 62. Hence, summarizing the function of exosome in IDD and property of exosome-based biotherapy is necessary.

## Exosome Derived from IVD Cells

### Structure and Function of Exosome

Exosome is a nano-sized substance which has spheroid membranes of a uniform lipid bilayer. It is a type of extracellular vesicle 63, which also includes microvesicles and apoptotic bodies 22. The diameter range of exosome are in the 30-150 nm; microvesicles are in the 200-1000 nm; apoptotic bodies are in the 800-5000 nm 64, 65. Average diameter of exosome is 100 nm, with the density ranging from 1.13 g/ml to 1.19 g/ml 66-69. It was first described by Stahl and Johnstone in the 1980s, as nano-sized vesicles discovered during reticulocyte maturation 17,70. At the earliest, it was hypothesized that the function of exosome was to eliminate unwanted proteins of cellular from cytoplasm 19. With further exploration in exosome, studies suggest that the function of exosome is potent and remarkable. It is not only the dumpster of cell, but could conduct the intercellular communication and response induction 21, 71.

Numerous types of cells could secrete exosome. It is detected in various body fluids, such as breast milk, cerebral spinal fluid, amniotic fluid, bile, saliva, semen, blood, lymph and amniotic fluid 72-77. Exosome is derived from endocytic pathway, in which the early secretory endosome was formed during the inward budding of the intracellular endsomal membrane, intracellular multivesicular bodies containing intraluminal vesicles are formed. With the maturation of the endosome, intraluminal vesicles were secreted as exosome by fusion with the plasma membrane 78, 79. Hitherto, studies reveal that exosome could secrete various bioactive molecules, such as mRNA, miRNA and proteins, and affect receptor cells by transmitting bioactive molecules 24. However, more studies are needed to explore the underlying mechanism of their roles.

#### Exosome Derived from NP

Increasing evidence implicates that NPc and NP stem cell-derived exosome participate in the process of IDD. Chen et al. took the IDD structure of rat caudal vertebra, re-cultured it in medium, thus utilizing IL-1β inducing senescence of NPc and isolating the senescence NPc-derived-exosome (SNPC-Exo) 80. They uncovered that incubated with SNPC-Exo made normal NPc evinced a degradation-related manifestation, particularly in the declined capacity of colony and proliferation. Besides, they revealed that SNPC-Exo mainly regulates and promotes the senescence of NPc by targeting the P53/P21 signaling pathway. All these results supported that SNPC-Exo might possess bioactive substances that derived from SNPC and play a key role in accumulating the senescence of normal NPc.

Various inflammatory and pre-inflammatory cytokines, such as IL-1β, IL-6, MMP-13 and TNF-α, participate in the process of IDD 81-86. Zhang et al. 87 discovered that degenerative NPc could secrete exosome which carried miR-16 and directly inhibited the anti-apoptotic IGF-1 / IGF-1R signaling pathway, thereby accumulating the apoptosis of NPc. This research demonstrated that degenerative NPc could affect the normal NPc by secreting exosome to withhold the anti-apoptotic pathway, leading to the senescence and degeneration of NPc.

Accumulating evidence implicated that NPc-derived-exosome (NPc-exo) not only could influence NPc, but exerting effect to CEP. Feng et al. 88 revealed that degenerative NPc-exo (dNPc-exo) could be taken up by CEP cells (CEPc), thereby decreasing the expression of Bcl-2, increasing the expression of Bax and Caspase-3, which are cell apoptosis makers. Thus, they elucidated that dNPc-exo could induce the apoptosis of CEPc. Also, they suggested that dNPc-exo could promote the degradation of ECM and the IDD process. Collectively, this study demonstrated the intercommunication between NPc-exo and CEPc, showing that all parts of IVD cells have intimate correlation.

Autophagy, as a catabolic self-digestion progression, could sustain the cellular homeostasis via dislodging dysfunctional organelle debris or hazardous macromolecules 89. In human chondrocytes, rapamycin could activate autophagy of articular chondrocytes, thereby promoting the secretion of extracellular vesicles 90. Zhang et al. 91 utilized rapamycin to accumulate the autophagy of NPc and found that the deliverance of NPc-exo was promoted. They further investigated that rapamycin-induced NPc-exo could carry miR-27a to target and inhibit MMP-13, thereby suppressing the degradation of ECM and delaying IDD progression. Also, they elucidated that autophagy could promote the secretion of NPc-exo via targeting the RhoC/ROCK2 pathway 92. This research provides a potential strategy for fabricating a vast amount of bio-synthesizing exosome.

Notochordal cells (NC) is the precursor cell of NPc. It appears in the embryonic stage and is gradually replaced by NPc during the development period in human. The remaining NC in the NP progressively disappeared after adolescence, and the degenerative process of IVD commenced 93. Our group 94 revealed that notochordal cell-derived exosome (NC-exo) could mitigate the vascularization process of IDD. We, for the first time, unearthed the NC-exo and demonstrated that NC-exo could alleviate angiogenesis via carrying high expressed miR-140-5p to endothelial cells, thus regulating the downstream Wnt/β-catenin pathway. Additionally, we suggested that, for NC, 0.5 MPa is a suitable mechanical condition for luring the secretion of NC exosomes and its capacity of anti-vascularization. Consequently, with a spectrum of experiments, we elucidated that NC-exo plays a pivotal role in the anti-angiogenesis effect of IVD and IDD progression.

Nevertheless, the specific biological mechanism of NPc-exo/NC-exo, its role in the process of IDD and its intercommunication with other IVD cells remains vague, needing further exploration and illustration.

#### Exosome Derived from AF

Recently, we 95 isolated AF-derived exosome (AF-exo) and stimulated the depravity of AF cell (AFc). With a spectrum of experimentations, we unveiled that degenerative AFc (dAFc) could secrete exosome, thus excreting pro-vascularization effect by promoting cell migration and inflammatory factor expression. Interestingly, the exosome secreted by non-degenerative AFc (ndAFc) could prevent blood vessels from growing in and retain the homeostasis of IVD. So, we manifested that ndAFc and dAFc derives different types of exosome and acts opposite roles in the degradation process of IVD. Nevertheless, there is still no study investigates about the underlying molecular mechanism of AF-exo function.

#### Exosome Derived from CEP

While investigation in CEP chondrocyte-derived exosome is scarce, studies have shown the existence of CEP stem cell-derived exosome (CESCs-exo).

Luo et al. 96 isolated and extracted the cartilage endplate stem cells (CESCs) and CESCs-exo. They revealed that CESCs-exo could promote the autophagy and withhold the apoptosis of NPc by activating PI3K/AKT/autophagy signaling pathway. Notably, they found that non-degenerative CESCs-exo is more effective than degenerative CESCs-exo. Besides, they figured out that non-degenerative CESCs-exo could impetus CESCs changing into NPc. CEP inflammation, however, influences this progression and aggravates IDD procession. Therefore, this study showed that CESCs-exo plays a striking role in the NPc degeneration. In the follow-up research, Luo et al. 97 found that CESCs-exo could activate HIF-1α/Wnt signaling pathway via autocrine mechanisms, thus promoting the secretion of TGF-β1 and GATA4. All of which suggested that CESCs-exo could accumulate the CESCs transforming into NPCs and delay the development of IDD. Chen et al. 98 detected that miR-125-5p, secreted by CESCs-exo, could target histone methyltransferase (SUV39H1), thereby promoting NPc autophagy, suppressing NPc apoptosis and delaying ECM degradation.

Collectively, all of these studies confirmed that CESCs-exo participates in the process of IDD. Thus, it is feasible to hypothesize that exosome derived from CEP cells (CEP-exo) also involves in IDD procession. Like AF-exo, non-degenerative CEP-exo and degenerative CEP-exo might play different roles: non-degenerative CEP-exo delays and reverses the process of IDD; degenerative CEP-exo induces and accelerates the process of IDD. However, this hypothesis needs further research to prove.

## Exosome Derived from Stem Cells

Stem cells, the most primitive cells at the top of the origin of cell lines, have multi-directional differentiation potential and self-renewal potency. They are abundant and easy to obtain, can proliferate in the low oxygen circumstances. Notably, mesenchymal stem cells (MSC), as a type of stem cells, has the ability of self-renewal and multi-directional differentiation 99-101. It was first isolated from bone marrow, and then unearthed in many tissues. For example: periosteum; muscle; placenta; fat; umbilical cord; umbilical cord blood; and other tissues 102-104. It has great therapeutic potential. In the field of IDD, the studies mainly focus on bone marrow mesenchymal stem cells (BMMSC), adipose-derived mesenchymal stem cells (ADSC), human placental mesenchymal stem cells (HPMSCs) and urine-derived stem cells (USCs).

### Exosome Derived from Bone Marrow Mesenchymal Stem Cells

BMMSC is derived from mesoderm and has multi-differentiation potential. Increasing lines of evidence have shown that BMMSC could be applied in curing diseases. For instance: spinal cord injury; bone regeneration; IDD; and so on. Nevertheless, it still has some unsolved problems, such as immunological reaction and potential tumorigenesis. Besides, severe environment of IDD also arrest BMMSC proliferation. Interestingly, BMMSC derived-exosome (BMMSC-exo) can resist the influence of harsh environment of IDD. Li et al. 105 cultured the NPc in different PH circumstances, and incubated it with BMMSC-exo. They revealed that, with the decreased of PH value, NPc and ECM showed a series of degradation: The proliferation of NPc was descended; the degradation of ECM was strengthened. Significantly, they discovered that BMMSC-exo could decelerate the apoptosis of NPC, promote the synthesis of chondrocyte ECM, and downregulate the matrix-degrading enzymes. All of which revealed that not only does MSC-derived-exosome (MSC-exo) have approximate function with MSC, it could also survive in abnormal biological ambience of IDD. This study elucidated that MSC-exo might be a better choice for biologic therapy.

Numerous of studies have explored the BMMSC-exo function and its communication with NPc and APc. Lu et al. 106 first illustrated the intercommunication between BMMSC-exo and NPc-exo. They unearthed that NPc-exo could promote the BMMSC migration and induce BMMSC differentiation to the NP-like phenotype. Additionally, they revealed that BMMSC-exo could promote NPc proliferation and ECM production in degenerative NP. This research proved the potent function of BMMSC-exo and its intercommunication with NPc. It also elucidated that NP could impact MSC via secreting NPc-exo. Hu et al. 107 isolated the BMMSC-exo and co-cultured it with NPc. They found that BMMSC-exo plays a prominent part in NPc apoptosis process which was induced by compression. BMMSC-exo could inhibit compression-induced NPc apoptosis by suppressing oxidative stress. Therefore, when develops the BMMSC-based injectable biological drug, considering its interrelate with IVD cells is pivotal.

BMMSC-exo and its target pathways have been explored extensively. Advanced glycation end products (AGEs) 108-110, formed by non-enzymatic reaction of reducing sugars with free amino groups of macromolecules, leads to ER stress, thus activating the unfolded protein response (UPR) 111, 112. Furthermore, UPR could initiate the C/EBP homologous protein, which is a responsible protein that modulates and induces cell apoptosis. Liao et al. 113 suggested that the endoplasmic reticulum (ER) stress markers and NPc apoptosis promoted in the process of IDD. They revealed that, under the AGEs stimulation, BMMSC-exo could alleviate ER stess-induced NPc apoptosis through AKT/ERK signaling pathway.

PI3K/AKT/mTOR signaling pathway is a pivotal regulator of autophagy 114, 115. Li et al. 116 revealed that BMMSC-exo could ameliorate the inflammation and apoptosis of AFc by inhibiting the expression of PI3K/AKT/mTOR signaling pathway.

Wen et al. 117 uncovered that BMMSC-exo which carried miR-199a could inhibit and reverse the process of IDD by targeting GREM1 and downregulating the TGF-β pathway. Zhu et al. 118 found that BMMSC-exo could alleviate the NPc degeneration and IDD progression by delivering miR-142-3p to target MLK3, thereby suppressing MAPK signaling pathway. Wang et al. 119 unveiled that BMMSC-exo could deliver miR-129-5p, target SOX4, inhibit the activation of Wnt/β-catenin pathway, thus promoting the proliferation of degenerative NPc and the synthesis of ECM. Zhu et al. 120 explored that BMMSC-exo could attenuate the apoptosis of NPc and degradation of ECM by carrying miR-532-5p to inhibit target RASSF5 pathway, which is a considerable apoptotic and/or senescence pathway.

However, most of studies about BMMSC-exo places emphasis on exploring its communication with NPc. More research is needed to explore BMMSC-exo correlation with AF and CEP cells.

### Exosome Derived from Adipose-Derived Mesenchymal Stem Cells

Despite accumulating evidence suggesting the benefits of BMMSC, it still has some limitation. For instance: expensive cost; collection difficulty; and the trauma to patient 121. Thus, it is essential to explore other types of MSC which could be collected and isolated easily. Notably, ADSC has received interest because of its easy access 122. ADSC and ADSC-derived exosome (ADSC-exo) could be applied in treating diseases and tissue damage, such as peripheral nerve injury, ischemic stroke and ruptured tendon 123-127. Xing et al. 128 supported that ADSC-exo could inhibit the release of NLRP3, thus affecting the pyroptosis of NPc. Also, it could alleviate the expression of MMPs, thereby blocking the catabolism of ECM. They constructed a thermosensitive acellular ECM hydrogel coupled with ADSC exosomes (dECM-exo), which will be discussed later.

The studies about the function of ADSC-exo in IDD procession still scarce. But the application of ASDC in treating other diseases could provide inspiration. Hepatic ischemia-reperfusion (I/R) injury is a complex procession which includes hypoxia, apoptosis, inflammatory mediator and lipid peroxidation 129, 130. In addition, GSK-3β could accumulate the expression of anti-apoptotic protein (Bcl-2 and survivin) in cells; ERK1/2 could induce the anti-apoptotic function by alleviating Bax protein and increasing of Bcl-2. Zhang et al. 131 revealed that ADSC-exo could carry PGE2, induce the inactivation of GSK-3β, upregulate ERK1/2, thus alleviating the secretion of inflammatory mediators and inhibiting the apoptosis of cells. Therefore, ADSC-exo might also carry PGE2, thereby alleviating the apoptosis of NPc. However, further research is needed to testify this hypothesis.

### Exosome Derived from Human Placental Mesenchymal Stem Cells

As known to all, BMMSC is gold standard when choosing MSC, whereas the hardship of obtaining BMMSC is a difficult problem 99, 121. Increasing evidence implicates that HPMSCs is easily obtained and ethically favored. Thus, it could be an alternative choice 132-134.

Pyroptosis, as an inflammatory cell death, is a pivotal mediator of inflammatory response 135. Yuan et al. 136 suggested that HPMSCs-derived exosome (HPMSCs-exo) could carry miR-26a-5p to inhibit METTL14/NLRP3 signaling pathway, which is a noticeable pathway interrelates with the pyroptosis and pro-inflammatory cytokines 137-139. Besides, HPMSCs-exo could alleviate the inflammatory conditions by suppressing cytokine release, thereby alleviating the pyroptosis of NPc.

ZNFs, the functional proteins related to the regulation of gene expression, are regulated by various types of miRNA 140. Accumulating studies suggested that ZNFs could regulate the cell proliferation. Wu et al. 141 supported that miR-1247 could directly repress ZNF346 expression, and thus inhibiting the progression of childhood neuroblastoma. Interestingly, increasing evidence implicates that ZNF121 has the capacity of regulating cell proliferation and apoptosis 142. Yuan et al. 143 unearthed that miR-4450 specifically targeted and inhibited the expression of ZNF121. Besides, they found that the knockdown of miR-4450 showed protective effect on NPc. Therefore, they elucidated that HPLMSC-exo could carry antagomiR-4450 to upregulate the expression of ZNF121, thereby alleviating the degradation of NPc.

### Exosome Derived from Urine-derived Stem Cells

USCs could be obtained from non-invasive sources, and have lower cost of culture and faster proliferative rate 144, 145. Interestingly, Qin et al. 145 suggested that USCs have longer telomere sequences and higher telomerase activity than other types of MSC, which is related to the proliferation ability. Thus, USCs is a promising source of exosome extraction and stem cell therapy.

MATN3 could promote IL-1ra expression and alleviate the IL-1β-induced catabolic matrix proteinases secretion 146. Guo et al. 147 discovered that USCs-exo could carry MATN3, thereby reversing the degradation of ECM and the process of IDD.

ER stress, which is mentioned above, could induce the apoptosis of NPc 148. Xiang et al. 149 identified that USCs-exo could inhibit the secretion of CHOP, GRP78, caspase-3 and caspase-12, thus inhibiting ER stress and the NPc apoptosis. They also revealed that USCs-exo could alleviate ER stress-induced apoptosis by activating the AKT and ERK signaling pathway.

## Exosome-based Therapeutic Strategy

Accumulating studies have detected that exosome plays a striking role in containing the homeostasis of IVD. For instance: increasing autophagy; inhibiting inflammatory response pathway; promoting the synthesis of ECM; and alleviating pyroptosis 122, 150. Hence, it is a better source for biological therapeutic strategy.

Xing et al. 128 developed an IVD biological hydrogel which is an ECM biological scaffolds loaded with exosome (dECM@exo) derived from ADSC. dECM, as an acellular scaffold, is a structure which loads the exosome. They uncovered that dECM@exo could slow the release of exosome while showing high load rate of exosomes. It is a regulator of inflammatory complexes and metalloproteinases. The combination of acellular scaffolds and exosome, both have low immunogenicity, makes exosome-based therapy be a better choice than cell-based therapy.

PI3K/AKT/mTOR signaling pathway is a vital regulator of autophagy 151. Luo et al. 152 revealed that Sphk2 could activate PI3K/AKT signaling pathway, thus promoting the autophagy of NPc and reversing the process of IDD. They encapsulated the CESCs overexpressing Sphk2 in an ECM of costal cartilage (ECM-Gels) and injected it near the CEP of rat. And then, they found that ECM-Gels could produce Sphk2-engineered exosomes which penetrated the AF and transported Sphk2 into NPc, activated the PI3K/AKT signaling pathway, thereby accumulating the autophagy of NPc.

Taken together, compared with others, exosomes have many preponderances, such as lower immune response and higher transfer efficiency 153. Thus, exosome-based therapeutic strategy has potent treatment potential and profound therapeutic implications, even further discovery is conducive to the development of it. Until now, some laboratories and companies have begun to investigate the engineered and mass-produced exosome. However, no exosome injection drugs for IDD have been approved for launch. The studies still stay in animal and preclinical experiment stage.

## Discussion

LBP, as a chronic and prevalent condition, gives great burden to social economy and quality of life 154, 155, which is mainly caused by IDD. Nevertheless, the current therapeutic strategies for IDD could not achieve satisfactory results. Hence, it is essential to pursue a new therapeutic strategy. Exosome, which plays a striking role in IDD progression, has got more and more attention. Accumulating evidence implicates that exosome participates in the degradation process of IVD. Therefore, when investigates the pathological changes of IVD, taking exosome into consideration is pivotal (Table 1).

As known to all, exosome-based drugs have great therapeutic potential. Nevertheless, it is essential to characterize the difficulties we faced. First, as a product secreted by various types of cells, exosome could be affected by multiple factors. For example: the source of cells; the state of cells; and the condition of culture. Secondly, IDD is an intricate process which includes manifold pathological changes. It is necessary to choose the exosome which aims at major pathological changes of IDD. Thirdly, as the largest avascular tissue in the body, the physiological condition of IVD is harsh. For instance: long-term internal high pressure; low pH; low nutrition; low oxygen; and complex inflammatory environment. All of which impact the activity and function of exosome 48. Last but not least, the accurate dose and injection position of exosome-based drugs are still unclear. Collectively, it is pivotal to even further explore specific function of exosome and precise dose of exosome-based injectable drugs.

## Conclusion

**Figure 1:**
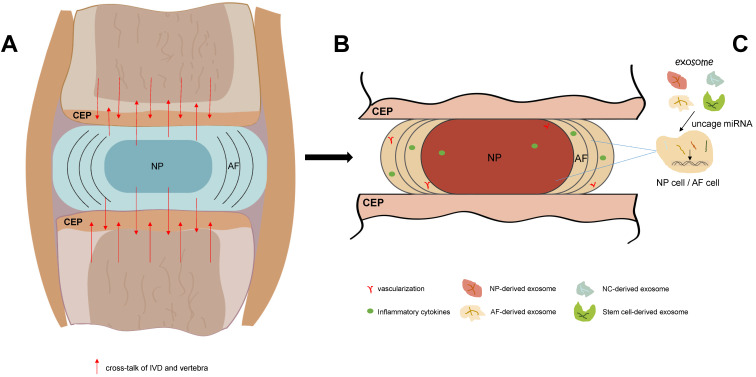
** The process of IDD and exosome-based therapeutic strategy. A.** In IVD, with the redundant mechanical load and other elements, the homeostasis of IVD is blemished. IVD also produce cross-talk which could influence the adjacent vertebrae. **B.** As the gradually boosts of IDD, vascular and nerves grows into IDD, and inflammatory cytokines cluster in IVD and surround tissues. **C.** Exosome that derived from non-stem cell or stem cell could carry various types of miRNA, implicate the proliferation and apoptosis of IVD cells, and thus delaying and reversing the IDD progression.

Exosome, as a substance which transmits information between cells, has attracted more and more attention. As a new direction for the therapeutic approach of IDD, exosome could influence IVD cells via various ways. For example: accumulating the autophagy; increasing the ECM synthesis; alleviating the apoptosis; and inhibiting the pyroptosis. Thus, it has remarkable potential to delay and reverse the onset and development of IDD. Further study is needed to explore the regulation mechanism of exosome, its intercommunication with IVD cells, and the safety/effectiveness of exosome-based therapeutic strategy (Figure 1).

## Figures and Tables

**Table 1:** Exosome and its targeting ways

Exosome derived from:	Carry	Target	Reference
SNPc	unknown	P53/P21	80
DNPc	miR-16	IGF-1/IGF-1R	87
RINPc	miR-27a	MMP-13	91
NC	miR-140-5p	Wnt/β-catenin	94
CESCs	unknown	PI3K/AKT/autophagy	96
CESCs	unknown	HIF-1α/Wnt	97
CESCs	miR-125-5p	SUV39H1	98
CESCs	Sphk2	PI3K/AKT	152
BMMSC	miR-199a	GREM1/ TGF-β	117
BMMSC	miR-142-3p	MLK3/ MAPK	118
BMMSC	miR-129-5p	SOX4 Wnt/β-catenin	119
BMMSC	miR-532-5p	RASSF5	120
BMMSC	unknown	AKT/ERK	113
BMMSC	unknown	PI3K/AKT/mTOR	116
ADSC	unknown	NLRP3	128
HPMSCs	miR-26a-5p	METTL14/NLRP3	136
HPMSCs	antagomiR-4450	ZNF121	143
USCs	MATN3	unknown	147
USCs	unknown	AKT/ERK	149

DNPc: degenerative NPc; RINPc: rapamycin-induced NPc.

## Abbreviations

LBP: low back pain
IVD: intervertebral disc
IDD: intervertebral disc degeneration
NP: nucleus pulposus
NPc: nucleus pulposus cells
AF: annulus fibrosus
AFc: annulus fibrosus cells
ECM: extracellular matrix
CEP: cartilaginous endplates
CEPc: cartilaginous endplates cells
TDR: total disc replacement
SC-Exo: stem cell-derived exosome
SNPC-Exo: senescence nucleus pulposus cells-derived-exosome
NPc-exo: nucleus pulposus cells-derived-exosome
dNPc-exo: degenerative nucleus pulposus cells-derived-exosome
NC: Notochordal cells
NC-exo: notochordal cell-derived exosome
AF-exo: annulus fibrosus-derived exosome
dAFc: degenerative annulus fibrosus cells
ndAFc: non-degenerative annulus fibrosus cells
CESCs-exo: CEP stem cell-derived exosome
CESCs: cartilage endplate stem cells
CEP-exo: exosome derived from cartilaginous endplates cells
MSC: mesenchymal stem cells
BMMSC: bone marrow mesenchymal stem cells
ADSC: adipose-derived mesenchymal stem cells
HPMSCs: human placental mesenchymal stem cells
USCs: urine-derived stem cells
BMMSC-exo: bone marrow mesenchymal stem cells derived-exosome
MSC-exo: mesenchymal stem cells-derived-exosome
AGEs: advanced glycation end products
UPR: unfolded protein response
ER: endoplasmic reticulum
ADSC-exo: adipose-derived mesenchymal stem cells-derived exosome
HPMSCs-exo: HPMSCs-derived exosome
dECM@exo: ECM biological scaffolds loaded with exosome
ECM-Gels: extracellular matrix of costal cartilage
